# Integrin Bidirectional Signaling: A Molecular View

**DOI:** 10.1371/journal.pbio.0020169

**Published:** 2004-06-15

**Authors:** Jun Qin, Olga Vinogradova, Edward F Plow

## Abstract

Cells receive and send signals across the plasma membrane using the integrin family of receptors. What is it about their structure that can mediate their function?

It goes without saying that the cellular plasma membrane effectively creates a barrier between the inside (intracellular area) and outside (extracellular area) of the cell it defines. In order for the cell to sense and respond to its environment (including other cells and the supporting structures that comprise the extracellular matrix [ECM]) and for the environment to influence cell function (including cell growth and movement), bidirectional signaling across the plasma membrane has to be mediated by receptors and other structures. About two decades ago, it became widely appreciated that many of the cell surface receptors that mediate cell–cell and cell–ECM interactions were structurally and functionally related, and the term “integrins” was coined to reflect the capacity of members of this family to integrate the extracellular and intracellular environment (Hynes 1987). Integrin-mediated interactions are vital to the maintenance of normal cell functioning because of their ability to mediate inside-out (intracellular to extracellular) and outside-in (extracellular to intracellular) signaling. Integrin dysfunctions are associated with numerous human disorders such as thrombosis, atherosclerosis, cancer, and chronic inflammatory diseases. Despite a total of nearly 30,000 integrin-related articles in the literature, intensive effort—more than 200 articles per month—continues to focus on understanding the roles of integrins in both physiological and pathological processes.

## The Integrin Family

**Figure 1:**
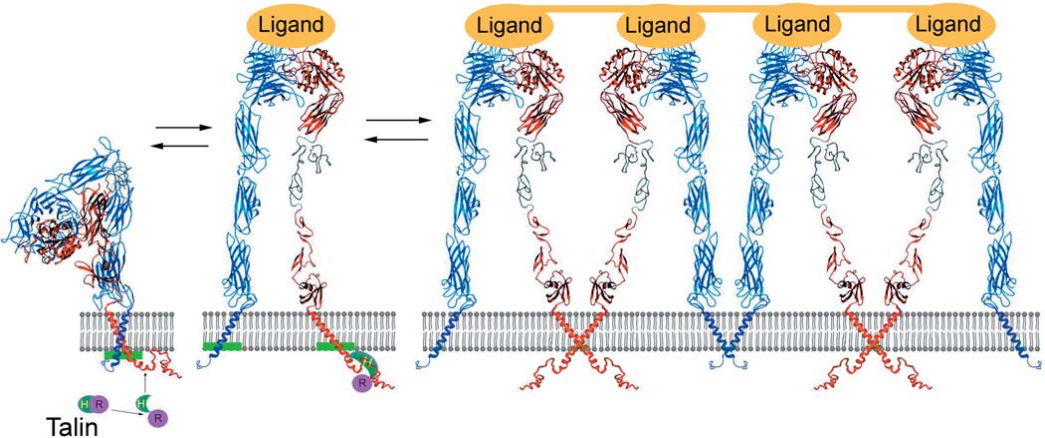
A Model for Integrin Inside-Out Activation and Clustering Cellular stimulation induces a conformational change in talin that exposes its talin head domain. The talin head domain binds to the β cytoplasmic tail, which displaces the α tail from its complex with the β tail, which in turn leads to an unclasping and a membrane-associated structural change of the cytoplasmic face (Vinogradova et al. 2002, 2004). Notice the proposed shifted membrane interface for both membrane-proximal helices before and after unclasping (green bars), which suggests a “fanning-out” unclasping process (Vinogradova et al. 2004). The unclasping initiates the opening of the integrin C-terminal stalks—including the transmembrane domains (Luo et al. 2004)—which is necessary for the switchblade shift of the extracellular headpiece from the bent to the extended form for high-affinity ligand binding (Takagi et al. 2002). The α subunit is in blue and the β subunit is in red. The ligated integrins cluster, possibly via oligomerization of transmembrane domains (Li et al. 2003). The model was generated based on the crystal structure of α_v_β_3_ extracellular domain (Xiong et al. 2001) and the nuclear magnetic resonance structure of the cytoplasmic domain (Vinogradova et al. 2002, 2004) with the helices extending to the transmembrane domain.

The integrin family comprises 20 or more members that are found in many animal species, ranging from sponges to mammals (Hynes 2002). They consist of two distinct, associated subunits (noncovalent heterodimers), where each subunit (α, β) consists of a single transmembrane domain, a large extracellular domain of several hundred amino acids (composed of multiple structural domains), and typically, a small cytoplasmic domain of somewhere between 20–70 residues (Figure 1). The extracellular domains bind a wide variety of ligands, whereas the intracellular cytoplasmic domains anchor to cytoskeletal proteins. In this manner, the exterior and interior of a cell are physically linked, which allows for bidirectional transmission of mechanical and biochemical signals across the plasma membrane, and leads to a cooperative regulation of cell functions, including adhesion, migration, growth, and differentiation. A central topic in the integrin research over the past decade has been the mechanism of inside-out activation (Liddington and Ginsberg 2002). In their resting state, integrins normally bind the molecules that activate them with low affinity. Upon stimulation, a cellular signal induces a conformational change in the integrin cytoplasmic domain that propagates to the extracellular domain. Integrins are transformed from a low- to a highaffinity ligand binding state. Such inside-out regulation of integrin affinity states is distinct from the outside-in signaling observed upon activation of most other transmembrane receptors (e.g., growth factor–growth factor receptor interactions), including integrins. The inside-out signaling protects the host from excessive integrin-mediated cell adhesion, which could, for example, lead to spontaneous aggregation of blood cells and have profound pathological consequences.

## The Heads and Tails of Inside-Out Signaling

Mutational studies provided the initial hints that disruption of the non-covalent clasp between α and β cytoplasmic tails is clearly the event within the structure of the integrin that initiates inside-out signaling. Point mutations in the α and β cytoplasmic tails that are near the membrane or deletion of either region result in constitutive activation of the receptor (O'Toole et al. 1991, 1994; Hughes et al. 1995). Mutating a single specific residue in the cytoplasmic tail of either subunit led to integrin activation, but a double mutation, which would have allowed retention of a salt bridge between the subunits, did not (Hughes et al. 1996)—suggesting that integrin inside-out activation is dependent upon regulation of the interaction between the two subunits. In support of this hypothesis, peptides corresponding to α and β cytoplasmic tails have been shown to interact with each other (Haas and Plow 1996). Since these original observations, there has been an intensive effort to understand the mechanism for regulation of integrin activation by the cytoplasmic region (for a recent review, see Hynes 2002). On the road toward this goal, Ginsberg and colleagues discovered that the head domain of a cytoskeletal protein—talin—plays a key role in binding to integrin β cytoplasmic tails and inducing integrin activation (Calderwood et al. 1999). Many other intracellular proteins bind to the α and β cytoplasmic tails (Liu et al. 2000), but the importance of talin in integrin activation is particularly convincing since it has been confirmed by multiple laboratories (Vinogradova et al. 2002; Kim et al. 2003; Tremuth et al. 2004) using various methods including overexpression and gene knockdown (siRNA) approaches (Tadokoro et al. 2003). In 2001, Springer and coworkers provided evidence for a model by which separation of the C-terminal portions of the α and β subunits results in inside-out activation. They showed that replacement of the cytoplasmic-transmembrane regions by an artificial linkage between the tails inactivates the receptor, whereas breakage of the clasp activates the receptor (Lu et al. 2001; Takagi et al. 2001). Shortly thereafter, the model gained direct and strong experimental support from a structural analysis in which the membrane-proximal helices of the two subunits were found to clasp in a weak “handshake” that could be disrupted by talin or constitutively activating mutations (Vinogradova et al. 2002). The model has been further verified by other biophysical studies (Kim et al. 2003) and extended to other integrins (Vinogradova et al. 2004). Since the membrane-proximal regions of integrin α and β cytoplasmic tails are highly conserved, the generalization of this signaling mechanism to all integrins was to be anticipated. A dynamic image of how such cytoplasmic unclasping occurs at the membrane surface can now be modeled (Figure 1) (Vinogradova et al. 2004).

## Straightening Out the Outside

On the extracellular side, ground-breaking insights were provided when the crystal structure of the extracellular domain of integrin α_v_β_3_ (the nomenclature identifies the particular α and β subunits) was determined (Xiong et al. 2001). In addition to the exquisite structural details, the overall conformation was surprisingly bent (Figure 1), which contrasted with structures revealed by the earlier electron micrographic studies that showed an extended, stalk-like structure (Weisel et al. 1992). Springer and coworkers used a series of biochemical/biophysical experiments to suggest that the bent structure represents an inactive form of integrin (Takagi et al. 2002), whereas activation induces a switchblade shift that converts the bent form to the extended form (Figure 1). A molecular picture has emerged for integrin insideout activation where a cellular signal induces the conformational change of talin exposing its head domain allowing it to bind to the integrin β cytoplasmic tail. This interaction unclasps the complex between the cytoplasmic tails, which then allows a conformational shift in the extracellular domain from a bent to a more extended form for high-affinity ligand binding (Figure 1) (Takagi et al. 2002).

The activated integrins may then undergo clustering whereby the transmembrane domain of each type of subunit (the α or β) interacts with itself—called homotypic oligomerization of the transmembrane domains (Figure 1) (Li et al. 2003). Ligand occupancy and receptor clustering initiates outside-in signaling that, in turn, regulates a variety of cellular responses (see below). The three steps in Figure 1 occur as part of a dynamic equilibrium, and perturbation of any step can shift the equilibrium, leading to transient, partial, or permanent integrin activation/inactivation depending on the extent of perturbation. For example, deletion of aIIb cytoplasmic tail completely removes the clasp and permanently activates the receptor (O'Toole et al. 1991), whereas a particular disease mutation may only impair the clasp and partially activate the receptor (Peyruchaud et al. 1997). While the model in Figure 1 is based on direct structural evidence for the cytoplasmic face (Vinogradova et al. 2002; Kim et al. 2003) and the extracellular domain (Takagi et al. 2002), the changes in the transmembrane region remained speculative. In this issue of *PLoS Biology*, Luo et al. (2004) provide what is, to our knowledge, the first experimental evidence for the transmembrane domain separation, an event suggested by the model shown in Figure 1. By selectively altering the residues that can interact with one another, the authors defined a specific transmembrane domain interface in resting α_IIb_β_3_ and showed that this interface is lost upon activation of this integrin. Backed by extensive structural and biochemical data on the integrin cytoplasmic/extracellular domains, this transmembrane domain study takes the next vital step toward a more complete understanding of the unclasping mechanism for integrin activation. Although the energy required for lateral separation of the transmembrane domains in membrane appears to be high, the third step in Figure 1 (clustering via transmembrane domain oligomerization) may compensate for it.

## Filling in the Pieces

Despite the molecular level of our understanding of integrin activation, a number of key questions remain unresolved. Although we know that the membrane-proximal clasp on the integrin cytoplasmic face controls the integrin activation, the distal side of either the α or β cytoplasmic tails may also play a role in integrin activation, since other mutations indicate that the C-terminal membrane distal region is important in regulating integrin activation via a mechanism that is yet unknown. Thus, the picture for the cytoplasmic face-controlled inside-out activation may be substantially more complicated than specified in Figure 1. There may exist other factors, such as negative regulators, in cells that bind to the cytoplasmic tails or their complex, and control the conformational change required for integrin activation. Also, there may be pathways other than the talin-mediated one that lead to integrin activation. Structures of the integrin cytoplasmic face bound to talin and the many other proteins known to bind to the cytoplasmic tails of integrins will undoubtedly provide further insights. In the transmembrane region, although there is ample evidence for heterodimeric transmembrane domain association (Adair and Yeager 2002; Schneider and Engelman 2003; Gottschalk and Kessler 2004; Luo et al. 2004) and dissociation upon integrin activation (Luo et al. 2004), a definitive structural view is missing. Some studies have proposed that homo-oligomerization is essential for inducing integrin activation (Li et al. 2003). However, the data provided by Luo et al. do not appear to support this model. On the extracellular side, while the C-terminal unclasping and separation of the cytoplasmic and transmembrane regions appears to relieve the structural constraint and may allow the unbending of the extracellular domain to attain the high-affinity ligand binding state (Takagi et al. 2002), a thorough molecular understanding of this process awaits high resolution structures of the intact receptor in inactive and active forms.

## What About Outside-In?

Upon the inside-out activation, integrins bind to specific extracellular matrix proteins. However, for the integrins to grip tightly to the extracellular matrix to mediate cell adhesion and migration, the integrin cytoplasmic domains must be anchored to the cytoskeleton (Giancotti and Ruoslahti 1999). This is achieved by “outside-in” signaling, i.e., when an integrin binds to the extracellular ligand, it clusters with other bound integrins, resulting in the formation of highly organized intracellular complexes known as focal adhesions that are connected to the cytoskeleton. The focal adhesions incorporate a variety of molecules, including the cytoplasmic domains of the clustered integrins, cytoskeletal proteins, and an extensive array of signaling molecules. The high local concentrations of these molecules facilitate cascades of downstream intracellular responses via protein–protein interactions, which are linked to the cytoskeleton as well as to complex intracellular signaling networks. Although many intracellular components involved in outsidein signaling have been identified, and much has been learned about various signaling pathways involved in outside-in signaling (Giancotti and Ruoslahti 1999), a molecular view of how the various events occur in time and space is still very uncertain. In particular, little structural insight has been obtained for early outside-in intracellular events following ECM–integrin binding, e.g., upon ECM engagement. How is the integrin cytoplasmic domain connected to the cytoskeleton? How is this connection regulated during cell adhesion and migration? The next wave of structural information may provide insights into these important and fertile areas of investigation.

## Abbreviation

ECM: extracellular matrix
